# Medical information seeking behavior of urban patients in Zhejiang Province, China: a cross-sectional study

**DOI:** 10.1186/s12889-022-14017-8

**Published:** 2022-08-21

**Authors:** Liyun Liu

**Affiliations:** School of Humanities and Management, Zhejiang Chinese Medical University, No. 260 Baichuan Street, Fuyang District, Hangzhou, 311402 China

**Keywords:** Health information seeking behavior, Online medical information seeking, Medical information source, Timing of seeking, China’s health information technology industry

## Abstract

**Background:**

Health information seeking behavior (HISB) is a prevalent research topic. However, little is known about sociodemographic factors of HISB in China. This study aimed to examine the HISB of urban patients in China and identify predictors of source preference, online information seeking, and the timing of online seeking.

**Methods:**

Based on the Health Information National Trends Survey (HINTS), this study conducted a cross-sectional survey of 1653 participants in different types of hospitals in 3 cities of different income levels within Zhejiang Province, China. Binary logistic regression analysis and multinomial logistic regression analysis were used to identify predictors of source preference, online medical information seeking, and the timing of online seeking for urban patients.

**Results:**

The offline was the primary source of medical information for 58.61% of adult urban patients, while 78.19% had ever sought medical information online. 36.81% of online medical information seekers sought information before the medical visit, 8.65% sought information after the visit, and 54.54% sought information before and after the visit. China’s urban patients with higher education levels, higher income levels, young, active in internet use, and living in high-income cities were more likely to be active online medical information seekers (using the internet as the primary source) and online medical information seekers (having ever sought medical information online). Except for gender and age, most sociodemographic characteristics were not significantly associated with the timing of online medical information seeking.

**Conclusions:**

Significant predictors of active online medical information seekers and online medical information seekers in China were almost the same. Regional economic development had a significant direct impact on medical information seekers. Most sociodemographic characteristics were not significantly associated with the timing of online medical information seeking. The findings of this study imply that China’s health information technology industry has Chinese characteristics.

## Background

As internet access has been increasing steadily worldwide, the internet has become an increasingly important source of health information for the population worldwide, especially for internet users [1, 2]. Online health information seeking (OHIS) has become a hot research topic in recent decades. HISB research in China started in 2005, while OHIS research publications have been growing since 2011 [3]. Most previous studies have been conducted in Western countries such as the U.S. [2], while the number of studies conducted in China has not been in the list of the top eight countries of publication on OHIS studies [4, 5].

The research question on the individual’s primary source of health information (online vs. offline) is essential to understand the structure of the health information market. Most prior studies have examined individuals’ OHIS behavior from various online source channels, including the internet in general, and specific ones such as social media, apps, websites [2, 6]. Prior research on individuals’ preference for offline and online health information sources in China [7–10] has not identified the factors that affect individuals’ preference for online and offline sources.

The research question of whether individuals have ever sought health information online is crucial to predict the target consumer of the online health information market. Very few previous studies have indicated the proportion of participants who have ever sought health information and the characteristics of health information seekers in China [7]. However, there is a lack of studies that identify the characteristics of Chinese online health information seekers.

The research question of when online health information seekers seek information (prior-to-visit vs. post-visit) is critically important for health websites/apps to understand the specific demand of their target consumers and identify their core service. Previous studies have primarily examined patients’ prior-to-visit online health information seeking behavior [11, 12] or post-visit online health information seeking behavior [13, 14]. However, little is known about the timing of online health information seeking in China. There is a lack of studies identifying predictors of the timing of online health information seeking in China and outside China.

This study aimed to address three research questions: (1) What is the primary health information source for Chinese? (2) Who is the target consumer of China’s online health information market? (3) When do Chinese online health information seekers seek information? This study intended to identify predictors of OHIS behavior in China. The findings provided insightful implications for health information carriers to increase their competitive edge by positioning the target consumer, improving customer service, etc.

## Literature review

### HISB

The information source is one of the major concerns of HISB research [15–17]. The health information sources used are usually divided into online sources and offline sources [18, 19]. The online source is primarily referred to as the internet, while the offline source is primarily referred to as mainstream media offline, friends or family, medical professionals, etc. [7, 18–20]. Previous studies have suggested that there might be some systematic differences in health information source preferences (i.e., online vs. offline). The differences might be influenced by the information type, such as general issues and specific health issues [20]. There might also be source preference differences between different countries. The majority of Americans prefer the internet as their primary source of general health information, while the vast majority of Chinese prefer the offline source (especially doctors) as their primary source of general health information [7].

Previous studies have used descriptive statistical results to present the source preference of health information, but knowledge about predictors of the source preference for online and offline sources was limited [7, 18–20]. Previous studies have examined the health information source channel used by rural residents [10], middle-aged urban residents [9], urban elderlies [21], college students [22] in China. However, these studies have used the question “Where do you usually obtain health information?” to measure source channel preference, and the respondents could choose more than one source channel. Little was known about predictors of source preference of health information in China. This study aimed to explore the primary source of health information for Chinese patients and identify the predictors of source preference of health information.

### OHIS

Most previous OHIS studies, especially studies using HINTS data, have used a binary variable (a yes-or-no question of internet health information seeking) to measure OHIS behavior [23, 24]. Some studies categorized those who answered “yes” in the question of whether they sought health information online as online health information seekers [25]. Some studies categorized those who sought health information from both solely online sources and a combination of the internet and other sources as online health information seekers, while those who only sought health information from offline sources as offline health information seekers [19].

As noted, individuals who reported that online sources were their primary source of health information were active online health information seekers. However, online health information seekers who had sought health information online might not use online sources as their primary source of health information. Therefore, the source preference of health information and whether they had ever sought health information online were two separated but closely related research questions.

The findings of predictors of OHIS remained mixed. Predictors of OHIS were divided into four categories: psychosocial factors, instrumental factors, contextual factors, and demographic factors [2]. Most previous OHIS studies conducted in China have focused on psychosocial factors and instrumental factors [26–29]. However, the research on the sociodemographic factors of Chinese online health information seekers is scarce.

### The timing of online health information seeking

Prior studies have suggested that people seek health information for various motivations, including deciding whether to have a medical visit, preparing for a visiting appointment, finding second opinions, and obtaining clarity and confirmatory information in greater depth after a medical visit [30–33]. Accordingly, it was concluded that people might seek health information before the visit, after the visit, or before and after the visit. Most prior research conducted in countries outside China demonstrated that more health information seekers sought information after the visit rather than before the visit [30, 34]. Despite the prevalence of research on HISB and OHIS, research on the timing of online health information seeking is limited.

Very few previous studies have explored the timing of online health information seeking, and most of them were qualitative studies or descriptive statistical analyses [30, 31, 35]. Previous studies have identified predictors of patients’ pre-visit online health information seeking [12] or predictors of patients’ post-visit online health information seeking [13, 14]. There is a lack of studies that identify predictors of the timing of online health information seeking both in China and outside China.

## Methods

### Study design and data collection

The HINTS is a cross-sectional survey of the U.S. National Cancer Institute to measure how American adults access and use health information. Based on China’s context, this study designed the survey questionnaire by making modifications to HINTS. Zhejiang Province has headquarters of several digital health companies (e.g., WeDoctor and Ding Xiang Yuan) and the headquarter of the Alibaba Group which has expanded its health information division. As Zhejiang Province played a leading role in China’s health information technology industry, this study conducted a cross-sectional survey on individuals in 3 cities with different income levels in Zhejiang Province.

Proportionate stratified sampling was the sampling method, and gross domestic product (GDP) per capita was the criterion to stratify city income levels. According to Zhejiang Statistical Yearbook 2019 [36], 5 cities were categorized as high-income cities (GDP per capita > USD15 000), 3 cities were categorized as medium-income cities (USD15 000 ≥ GDP per capita ≥ USD10 000), and 3 cities were categorized as low-income cities (GDP per capita < USD10 000). The sample size of each income-level city stratum was proportional to the stratum size in relation to the total urban population of Zhejiang Province in 2018 [36]. The sampling weight of high-income cities was 52.72%, that of medium-income cities was 24.24%, and that of low-income cities was 23.04%.

### Participants and sample selection

A pretest with a sample of 39 participants was conducted in April 2019. This study conducted an in-person structured questionnaire survey in different types of hospitals (tertiary 3A, 3B, secondary 2A, 2B, and primary) in May and June 2019. A total of 1653 participants were randomly surveyed in the outpatient center of each hospital, and health practitioners were excluded from the survey. Hangzhou was a high-income city (*n* = 871), Jinhua was a medium-income city (*n* = 401), and Lishui was a low-income city (*n* = 381).

**Fig. 1:**
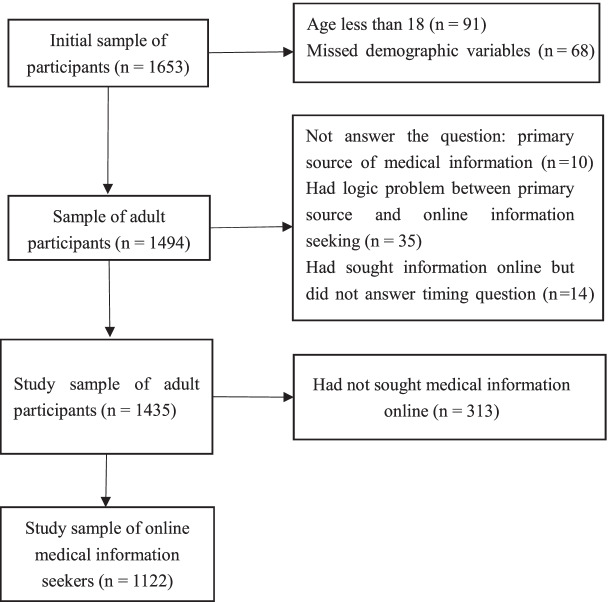
Flowchart of sample selection

Figure 1 presents the flowchart of sample selection. Among adult participants (*n* = 1494), the participants who did not answer the question of the primary source of medical information (*n* = 10) were excluded. Participants who self-reported that the internet was the primary source but self-reported that they never sought medical information online (*n* = 35) were excluded. Participants who self-reported that they had ever sought medical information online but did not answer the question on the timing of online medical information seeking (*n* = 14) were excluded. The study sample for the research question of source preference and online information seeking was *n* = 1435. Participants who had not ever sought medical information online were excluded, and the study sample of online medical information seekers was *n* = 1122. It was the study sample for the research question of the timing of online seeking.

### Measurement

#### Sociodemographic characteristics

Sociodemographic characteristics included gender, age, education level, marital status, monthly income, and self-rated health status. Monthly income levels were divided into low-income (less than 1800 CNY), medium-low income (1801—4600 CNY), medium-income (4601—8000 CNY), medium-high-income (8001—17,000 CNY), and high-income (more than 17,000 CNY) groups. Participants were asked to rate their current health status using a 5-point Likert scale, ranging from very good (coded as 1) to very poor (coded as 5).

#### Internet use

According to *the Internet Development Report of Zhejiang Province 2019* [37], 80.9% of its residents had internet access. Therefore, this study modified the HINTS question measuring internet use by asking: “Is the internet your primary source of information?” Those participants answering ‘yes’ were subsequently referred to as active internet users.

#### Primary source of medical information

This study measured the primary source of medical information by asking: “Which source is your primary source of medical information?” Participants had two options: one was the online source (mainly referred to the internet), and the other was the offline source. Previous studies found that the percentage of source preference for traditional media such as TV, magazines, and books was low [7, 18]. Therefore, the offline source hereafter was referred to doctors, family, relatives, and friends. Those who chose the internet as their primary source of medical information were subsequently referred to as active online medical information seekers.

#### Online medical information seeking

This study modified HINTS questions concerning online medical/health information seeking by asking, “Have you used the internet to look for medical information before?” Those who answered ‘yes’ were subsequently referred to as online medical information seekers.

#### The timing of online medical information seeking

This study used the question “When do you usually seek medical information online?” to measure the timing of online medical information seeking. There were three options. One was before the medical visit, the second was after the medical visit, and the third was before the visit and after the visit.

### Statistical analyses strategy

This study used Stata 12.0 software to conduct statistical analyses. Binary logistic regression analysis was used to identify the factors that affect adult patients’ medical information seeking behavior (primary source of medical information, online medical information seeking). Multinomial logistic regression analysis was used to examine the association of the patient characteristics with the timing of online medical information seeking.

## Results

### Sociodemographic characteristics

Table 1 shows the sociodemographic characteristics of the study sample. 59.44% of respondents were females. A total of 61.67% of respondents had a college graduate education level. Respondents aged 18–55 accounted for 94.01%. On the one hand, there was urban-rural education inequality in China [38], while the rural population was not included in this survey. On the other hand, the survey was conducted in Zhejiang Province, and its cities usually had a high stock and inflow of well-educated young people. A total of 85.09% of respondents were in good or fair health status. A total of 73.31% of respondents were active internet users.

**Table 1:** Descriptive statistics of the study sample (*n* = 1435)

Variable	n	%
Gender		
Male	582	40.56
Female	853	59.44
Age (year)		
18–29	603	42.02
30–40	515	35.89
41–55	231	16.10
56–65	56	3.90
> 65	30	2.09
Health status		
Very good	148	10.31
Good	586	40.84
Fair	635	44.25
Poor	62	4.32
Very poor	4	0.28
Marital status		
Married	882	61.46
Single	521	36.31
Divorced	27	1.88
Widowed	5	0.35
Education		
≤Primary school	58	4.04
Junior high school	182	12.68
Senior high school	228	15.89
College graduate	885	61.67
Postgraduate	82	5.72
Monthly income (CNY)		
≤ 1800	189	13.17
1801–4600	463	32.27
4601–8000	434	30.24
8001–17,000	246	17.14
> 17,000	103	7.18
Internet use		
Inactive user	383	26.69
Active user	1052	73.31
Primary source of information		
Offline source	841	58.61
Online source	594	41.39
Online information seeking		
No	313	21.81
Yes	1122	78.19

### Primary source of medical information

Table 1 shows that 58.61% of respondents self-reported that their primary medical information source was the offline. Table 2 presents the binary logistic regression results of medical information source preference. The primary source of medical information variable was used as the outcome variable, in which the offline source was coded as 0 and the online source was coded as 1.

**Table 2:** Logistic regression results of the primary source of medical information (base outcome = offline source, *n* = 1435)

Characteristics	OR^a^	95% CI^b^	***p***-value
Gender (Ref^c^: Male)	1.05	(0.82–1.33)	0.72
Age (Ref: 18–29)			
30–40	1.06	(0.76–1.49)	0.73
41–55	0.70	(0.45–1.10)	0.12
56–65	0.41	(0.17–0.98)	**0.05**
> 65	0.36	(0.10–1.31)	0.12
Marital status (Ref: Married)			
Single	1.17	(0.84–1.65)	0.35
Divorced	1.43	(0.59–3.45)	0.43
Widowed	0.47	(0.04–5.20)	0.54
Education (Ref: ≤ Primary school)			
Junior high school	1.91	(0.72–5.07)	0.20
Senior high school	2.64	(1.01–6.85)	**0.05**
College graduate	2.90	(1.14–7.38)	**0.03**
Postgraduate	4.44	(1.56–12.63)	**0.01**
Monthly income (Ref: ≤ 1800 CNY)			
1801–4600 CNY	1.17	(0.78–1.74)	0.45
4601–8000 CNY	1.15	(0.77–1.73)	0.49
8001–17,000 CNY	1.72	(1.10–2.71)	**0.02**
> 17,000 CNY	1.82	(1.03–3.24)	**0.04**
Health status	1.05	(0.90–1.23)	0.54
Internet use (Ref: Inactive)	2.31	(1.71–3.13)	**< 0.001**
City (Ref: Hangzhou)			
Jinhua	0.71	(0.52–0.97)	**0.03**
Lishui	0.41	(0.29–0.57)	**< 0.001**
Hospital type (Ref: Primary)			
Secondary B	0.89	(0.42–1.90)	0.77
Secondary A	0.77	(0.39–1.53)	0.46
Tertiary B	0.97	(0.55–1.72)	0.91
Tertiary A	1.15	(0.68–1.95)	0.59
Constant	0.11	(0.03–0.38)	**< 0.001**

^a^*OR* odds ratio

^b^*CI* confidence interval

^c^*Ref* reference group

Respondents aged 56–65 were less likely than those aged 18–29 to use the online as their primary medical information source (OR 0.41, 95% CI 0.17–0.98). Compared to respondents with primary school education and below, respondents with senior high school education (OR 2.64, 95% CI 1.01- 6.85), college graduates (OR 2.90, 95% CI 1.14–7.38), and postgraduates (OR 4.44, 95% CI 1.56–12.63) were more likely to use the online as their primary source of medical information. Compared to respondents with low monthly income, respondents with medium-high income levels (OR 1.72, 95% CI 1.10–2.71) and high-income levels (OR 1.82, 95% CI 1.03–3.24) were more likely to use the online as their primary source of medical information. Active internet users were more likely than inactive internet users to use the online as their primary source of medical information (OR 2.31, 95% CI 1.71–3.13). Compared to the respondents in high-income cities, respondents in medium-income cities (OR 0.71, 95% CI 0.52–0.97) and low-income cities (OR 0.41, 95% CI 0.29–0.57) were less likely to use the online as their primary source of medical information.

### Online medical information seeking

Table 1 shows that 78.19% of respondents had sought medical information online. Table 3 presents the logistic regression results of online medical information seeking. Online medical information seeking was the outcome variable in which the option “No” was coded as 0, while “Yes” was coded as 1.

**Table 3:** Binary logistic regression results of online medical information seeking (*n* = 1435)

Characteristics	OR^a^	95% CI^b^	***p***-value
Gender (Ref^c^: Male)	1.43	(1.04–1.96)	**0.03**
Age (Ref: 18–29)			
30–40	1.05	(0.65–1.69)	0.85
41–55	0.70	(0.40–1.24)	0.22
56–65	0.17	(0.07–0.39)	**< 0.001**
> 65	0.12	(0.04–0.39)	**< 0.001**
Marital status (Ref: Married)			
Single	0.95	(0.59–1.54)	0.85
Divorced	1.62	(0.60–4.35)	0.34
Widowed	0.33	(0.03–3.40)	0.35
Education (Ref: ≤ Primary school)			
Junior high school	2.94	(1.29–6.69)	**0.01**
Senior high school	6.05	(2.64–13.86)	**< 0.001**
College graduate	8.73	(3.87–19.68)	**< 0.001**
Postgraduate	35.47	(6.97–180.55)	**< 0.001**
Monthly income (Ref: ≤1800 CNY)			
1801–4600 CNY	1.45	(0.91–2.32)	0.12
4601–8000 CNY	1.70	(1.03–2.81)	**0.04**
8001–17,000 CNY	3.27	(1.71–6.27)	**< 0.001**
> 17,000 CNY	1.63	(0.73–3.64)	0.23
Health status	1.01	(0.83–1.22)	0.96
Internet use (Ref: Inactive)	2.35	(1.69–3.26)	**< 0.001**
City (Ref: Hangzhou)			
Jinhua	0.61	(0.41–0.91)	**0.02**
Lishui	0.47	(0.31–0.71)	**< 0.001**
Hospital type (Ref: Primary)			
Secondary B	1.01	(0.36–2.84)	0.98
Secondary A	1.65	(0.58–4.75)	0.35
Tertiary B	0.72	(0.30–1.69)	0.44
Tertiary A	1.03	(0.45–2.34)	0.95
Constant	0.28	(0.07–1.08)	**0.06**

^a^*OR* odds ratio

^b^*CI* confidence interval

^c^*Ref* reference group

Females were more likely than males to seek medical information online (OR 1.43, 95% CI 1.04–1.96). Compared to respondents aged 18–29, those aged 56–65 (OR 0.17, 95% CI 0.07–0.39) and older than 65 (OR 0.12, 95% CI 0.04–0.39) were less likely to seek online medical information. Compared to respondents with a primary school education level and below, respondents with a junior high school education (OR 2.94, 95% CI 1.29–6.69), senior high school education (OR 6.05, 95% CI 2.64–13.86), college graduate education (OR 8.73, 95% CI 3.87–19.68), and postgraduate education (OR 35.47, 95% CI 6.97–180.55) were more likely to seek medical information online. Compared to respondents with a low-income level, respondents with a medium-income level (OR 1.70, 95% CI 1.03–2.81) and respondents with a medium-high income level (OR 3.27, 95% CI 1.71–6.27) were more likely to seek medical information online. Active internet users were more likely to seek medical information online (OR 2.35, 95% CI 1.69–3.26). Compared to respondents in high-income cities, respondents in medium-income cities (OR 0.61, 95% CI 0.41–0.91) and low-income cities (OR 0.47, 95% CI 0.31–0.71) were less likely to seek medical information online.

### The timing of online seeking

Among 1122 online medical information seekers, 36.81% (413/1122) sought medical information before the medical visit, 8.65% (97/1122) sought information after the visit, and 54.54% (612/1122) sought information before and after the medical visit. Table 4 presents the multinomial logistic regression results of the timing of online medical information seeking. Seeking medical information online before and after the medical visit was the base outcome of the multinomial logistic regression model.

**Table 4:** The multinomial logistic regression results of the timing of online medical information seeking. (Base outcome = before and after the visit, *n* = 1122)

Characteristic	Before the visit			After the visit		
	RRR^a^	***p***-value	95% CI^b^	RRR	***p***-value	95% CI
Gender (Ref^c^: Male)	0.68	**0.01**	(0.51–0.89)	0.53	**0.01**	(0.33–0.86)
Age (Ref: 18–29)						
30–40	0.79	0.23	(0.54–1.16)	1.07	0.84	(0.56–2.06)
41–55	0.92	0.73	(0.56–1.51)	0.65	0.36	(0.26–1.63)
56–65	1.13	0.87	(0.28–4.48)	9.03	**0.01**	(2.18–37.33)
> 65	4.50	0.19	(0.47–43.44)	N/A^d^	1.00	N/A
Health status	0.88	0.16	(0.73–1.05)	1.11	0.51	(0.81–1.53)
Marital status (Ref: Married)						
Single	1.14	0.50	(0.78–1.66)	1.22	0.55	(0.63–2.35)
Divorced	1.14	0.79	(0.45–2.88)	N/A	0.99	N/A
Widowed	N/A	0.99	N/A	1.05	1.00	N/A
Education (Ref: ≤ Primary school)						
Junior high school	2.36	0.25	(0.55–10.20)	2.54	0.42	(0.26–24.52)
Senior high school	2.21	0.27	(0.53–9.20)	2.57	0.40	(0.28–23.58)
College graduate	1.56	0.53	(0.39–6.29)	0.96	0.97	(0.11–8.52)
Postgraduate	0.80	0.77	(0.18–3.54)	0.36	0.42	(0.03–4.30)
Monthly income (Ref: ≤ 1800 CNY)						
1801-4600CNY	0.67	0.09	(0.42–1.07)	1.10	0.83	(0.47–2.56)
4601-8000CNY	0.76	0.26	(0.48–1.22)	1.20	0.68	(0.51–2.83)
8001–17,000 CNY	0.72	0.21	(0.44–1.20)	0.71	0.50	(0.27–1.90)
> 17,000 CNY	0.63	0.17	(0.33–1.22)	1.38	0.56	(0.46–4.18)
Internet use (Ref: Inactive)	1.10	0.59	(0.78–1.56)	0.66	0.13	(0.39–1.13)
City (Ref: Hangzhou)						
Jinhua	1.19	0.33	(0.84–1.70)	1.38	0.30	(0.76–2.50)
Lishui	1.35	0.12	(0.92–1.97)	1.16	0.67	(0.60–2.24)
Hospital type (Ref: Primary)						
Secondary B	0.68	0.38	(0.28–1.61)	0.41	0.22	(0.10–1.68)
Secondary A	0.70	0.36	(0.32–1.50)	0.46	0.22	(0.13–1.59)
Tertiary B	1.02	0.96	(0.52–1.99)	0.61	0.35	(0.22–1.73)
Tertiary A	0.74	0.34	(0.41–1.37)	0.57	0.23	(0.23–1.43)
Constant	1.08	0.93	(0.21–5.39)	0.28	0.32	(0.02–3.47)

^a^*RRR* relative risk ratio

^b^*CI* confidence interval

^c^*Ref* reference group

^d^*N/A* not applicable

Most sociodemographic characteristics were not significantly associated with the timing of online medical information seeking. Compared to seeking medical information online before and after the visit, females were less likely to seek medical information online before the visit (RRR 0.68, 95% CI 0.51- 0.89) and after the visit (RRR 0.53, 95% CI 0.33- 0.86). Compared to seeking medical information online before and after the visit, respondents aged 56–65 were more likely than those aged 18–29 to seek medical information online after the visit (RRR 9.03, 95% CI 2.18- 37.33).

## Discussion

### The paradox of source preference, online seeking, and timing of seeking

This study indicated that the offline was the primary source of medical information for more than half of Chinese urban patients. It was consistent with the finding of a previous study that used the China 2017 HINTS data [7]. However, Guangzhou Community Health Survey 2013 showed that 26.2% of the urban population used health practitioners as their primary source of health information [39]. The share of the internet option was the highest for female adults [40] and middle-aged urban residents in eastern China [9] when they could choose more than one health information source channel. Therefore, the findings on health information source preference in China were inconsistent. The inconsistency could be explained by the differences in where and when the survey was conducted, who was surveyed, and how the question was measured. Despite the inconsistency in China, previous studies conducted in many other countries like the U.S. indicated that the internet was their primary source of health information [7, 20, 41].

This study also indicated a difference in the timing of medical information seeking between China and many other countries. More than half of Chinese patients (54.54%) sought medical information online before and after the medical visit. It was concluded that most Chinese had sought medical information online before the medical visit (91.35%) rather than after the visit (63.19%). However, more online health information seekers in foreign countries sought information after the visit rather than before the visit [30, 34].

Despite the differences in source preference and the timing of information seeking, there was no significant difference in online health information seeking. It was found that in each HINTS survey year (2007–2018), the majority of the U.S. adults (a range of 69.8%–81.5%) had ever sought health or medical information, and 68.9% across survey years reported using the internet first in their most recent health information seeking [42]. This study found that 78.19% of urban adults had ever sought medical information online. A cross-sectional survey conducted on rural adults in Zhejiang Province in 2015 showed that 40.60% of internet users had sought health information via the internet during the past year [43]. This inconsistency could be explained by the difference in when the survey was conducted and who was surveyed. This study indicated a paradox in source preference, online medical information seeking, and the timing of online seeking between China and many other countries.

### The special characteristics of China’s health information technology industry

The paradox of findings on source preference, online information seeking, and the timing of seeking, could be explained by the special characteristics of China’s health information technology industry. There are structural differences in the health information technology industry in China and Western countries. Website/app/WeChat public accounts are three primary modalities through which China’s health-related entities deliver their online health services to their consumers. China’s health websites/apps/WeChat public accounts primarily provide online service types such as health information and knowledge, in-person medical visit appointment scheduling, virtual consultation, and virtual visits.

Virtual consultations differ from virtual visits in service scope and health coverage in China [44]. China’s direct-to-consumer telemedicine development lagged behind many Western countries, especially the United States. Only after the National Health Commission of China issued specific regulation schemes for online medical diagnosis and treatment (the equivalent of virtual visits) and internet hospitals in 2018 [45], did virtual visits and internet hospitals grow tremendously. Before the issuing of the regulation schemes, virtual consultation was one of the most prevalent service types of direct-to-consumer telemedicine in China. The Communication Administration-licensed health websites/apps deliver virtual consultation services to users. However, they are not allowed to diagnose and prescribe treatments for patients. The Health Commission-licensed internet hospitals and virtual visit service providers deliver virtual visit services for non-first-visit patients.

With the help of telemedicine technology, patients can consult with the physician virtually and visit the physician virtually. Consequently, the online source was more likely to become the primary source of health information for internet users in Western countries such as the United States. Due to the development lag of virtual consultations and virtual visits, the offline source was still the primary source of health information for Chinese internet users. Meanwhile, as the offline source (primarily referred to as doctors) was the primary source of health information, in-person visits were the essential way for Chinese patients to obtain medical information.

There were structural differences in the health website/app service composition between China and Western countries. In-person visit online appointment scheduling was the core service type delivered by almost all medical websites/apps/WeChat public accounts in China. Due to the absence of an effective referral system, Chinese patients usually bypassed primary hospitals and directly accessed higher-level hospitals [46]. Previous research has also found that Chinese patients had the highest awareness and usage of in-person medical visit appointments and medical fee payments, among all types of online health information services [47]. The finding that 91.35% of Chinese patients had sought medical information before the visit indicated that preparing for a visiting appointment was one of the most important motivations for China’s online medical information seekers.

In addition to providing in-person visit online appointment scheduling, most of China’s medical websites/apps also provided health/medical information and knowledge for laypersons. However, the health/medical information most of China’s medical websites/apps/WeChat public accounts offered was much less comprehensive and systematic than those of their foreign counterparts, since many of their counterparts provided medical encyclopedia for laypersons according to health topics. Very few of China’s health websites provided medical encyclopedias for their consumers. Comprehensive and systematic health information and knowledge primarily satisfied the consumer’s need to expand knowledge and improve the understanding of the information received after a medical visit and the symptom checks before the medical visit. The underpinning role of in-person visit online appointment scheduling and the limited provision of health/medical information and knowledge have demonstrated that most of China’s health website/app service composition primarily aimed to solve the problems of in-person visit accessibility and convenience.

The abovementioned structural differences could explain the paradox of source preference, online information seeking, and the timing of online seeking between China and other countries. Due to the development lag of virtual visits and virtual consultations, the offline source was still China’s patients’ primary source of medical information, and in-person visits were still the essential way for them to access medical information. In-person visit online appointment scheduling was consequently a core service of most of China’s health websites/apps. In-person visit online appointment scheduling empowered Chinese patients to access the in-person visit conveniently, so the proportion of Chinese who had ever sought medical information online was very close to that of the United States. However, China’s online health information seekers’ motivations differed from the U.S. seekers. The United States’ online health information seekers had various motivations, such as preparing for the visit and expanding the understanding of the information received after the visit. China’s health information seekers went online primarily to prepare for an in-person visit appointment.

### Predictors of online medical information seeking behavior

This study indicated that China’s adults with a higher education level, higher income level, young, active in internet use, living in high-income cities were more likely to be active online medical information seekers (using the online as their primary source of medical information) and online medical information seekers (having ever sought medical information online). Most predictors were consistent with the findings of prior research. A systematic literature review and network analysis identified that the most central predictors of HISB were education, age, gender, financial income, and health condition [15]. A meta-analysis found that internet use and demographic factors such as age, gender, education, income, and race were significant predictors of OHIS [2].

Individuals with higher income and education levels usually had higher demands for high-quality and diverse health information and services; therefore, they were more likely to be active online medical information seekers and online medical information seekers. Information technology, including health information technology, was usually more friendly to young generations and active internet users; therefore, they were more likely to be active online medical information seekers and online medical information seekers. Females were more likely to be family caregivers [48], and therefore, they were more likely to seek health information online for themselves and their family members.

This study indicated that China’s patients in medium-income and low-income level cities were less likely to be active online medical information seekers and online medical information seekers. By dividing the literature into two subgroups of high-income countries/areas and low-income countries/areas, Zhao et al. used a meta-analysis to confirm that national economic development level differences had a moderating effect on users’ adoption behavior of online health [49]. Lu et al. also confirmed the differences in health information seeking behaviors and source preferences between China population and the U.S. population [7]. However, few previous studies have examined the direct effect of regional economic development differences on health information seeking behavior.

The significant direct impact of regional economic development on health information seeking behavior showed that the development of the online health information technology industry was limited by national/regional economic development. The literature indicated that a wider economic environment was one of the factors associated with the effective implementation of large-scale health information technology [50]. Compared with high-income regions, low-income and medium-income regions were less likely to have sufficient financial resources to develop health information technologies.

This study indicated that most sociodemographic factors were not significantly associated with the timing of online medical information seeking. Females were more likely to seek medical information online before and after the visit. As females were more likely to be family caregivers, they were more likely to prepare for the visiting appointment and expand their knowledge of the treatment and the care for themselves and their family members. Individuals aged 56–65 were more likely than those aged 18–29 to seek medical information online after the visit. One possible explanation was that they were more likely to have information overload problems during the visit, so they needed to expand their understanding of the information received after the visit.

### Limitations and future studies

This study has several limitations. First, as the survey was conducted in cities of Zhejiang Province, it was not a nationally representative survey. There are significant provincial differences in China, and I will further the study by conducting surveys in provinces with different income levels in the future. Second, there are significant urban-rural divide in China, and I will extend the research by surveying the rural population. Third, the survey was conducted before the outbreak of COVID-19, while the outbreak brought structural changes to China’s health information industry and consumer behavior toward virtual visits and virtual consultations. Future research should measure the effect of the outbreak of COVID-19 on the direct-to-consumer telemedicine development and health information seeking behavior in China.

## Conclusion

As a vast majority of respondents were online health information seekers and less than half were active online health information seekers, China’s online health information market was a burgeoning market. More Chinese online medical information seekers sought information before the visit rather than after the visit. This study indicated a paradox of medical information source preference, online seeking, and the timing of online seeking between China and other countries. The findings of this study imply that China’s health information industry has Chinese characteristics and is different from other countries.

Urban patients with a higher education level, higher monthly income level, active in internet use, young, and living in high-income cities were target consumers of the online health information market in China. China’s health information carriers could increase their competitive edge by positioning the target consumer. The outbreak of COVID-19 tremendously increased the awareness and actual use of virtual consultations and virtual visits in China, and the internet was increasingly becoming the primary source of health information for patients. Health websites/apps should maintain their advantages in in-person visit online appointment scheduling and prepare for the incoming structural change of the health information industry by improving their service composition.

## Data Availability

The datasets generated and/or analyzed during the current study are not publicly available due the terms of consent/assent to which the participants agreed but are available from the corresponding author on reasonable request.

## Abbreviations

HISB: Health Information Seeking Behavior
OHIS: Online Health Information Seeking
HINTS: Health Information National Trends Survey
